# Predictors of Procedural Success in Patients With Degenerated Surgical Valves Undergoing Transcatheter Aortic Valve-in-Valve Implantation

**DOI:** 10.3389/fcvm.2021.718835

**Published:** 2021-09-24

**Authors:** Abdelrahman I. Abushouk, Omar Abdelfattah, Anas Saad, Toshiaki Isogai, Medhat Farwati, James Yun, Zoran Popovic, Shashank Shekhar, Rishi Puri, Grant W. Reed, Amar Krishnaswamy, Samir R. Kapadia

**Affiliations:** ^1^Department of Cardiovascular Medicine, Heart, Vascular and Thoracic Institute, Cleveland Clinic Foundation, Cleveland, OH, United States; ^2^Department of Internal Medicine, Morristown Medical Center, Atlantic Health System, Morristown, NJ, United States; ^3^Department of Thoracic and Cardiovascular Surgery, Heart, Vascular and Thoracic Institute, Cleveland Clinic Foundation, Cleveland, OH, United States

**Keywords:** aortic stenosis, transcatheter, TAVI, valve-in-valve, procedural success

## Abstract

**Background:** Valve-in-Valve transcatheter aortic valve implantation (ViV-TAVI) is a growing alternative for redo-surgery in patients with degenerated surgical valves. To our knowledge, data are lacking on the determinants on ViV-TAVI procedural success in patients with degenerated surgical valves.

**Methods:** All consecutive patients undergoing ViV-TAVI for degenerated surgical valves at the Cleveland Clinic were analyzed. Data were extracted from our patient registry on baseline patient characteristics, echocardiographic parameters, and procedural details. To identify possible predictors of ViV-TAVI procedural success, we employed a multivariate logistic regression model.

**Results:** A total of 186 patients who underwent ViV-TAVI were analyzed, with procedural success (VARC-2 device success and absence of periprocedural MACCE) reported in 165 (88.7%) patients. Patients with successful ViV-TAVI were significantly younger and had more frequent utilization of the transfemoral access than those with failed procedure. Other baseline and procedural characteristics were comparable between both groups. In terms of echocardiographic parameters, the procedural success group had a significantly lower AV peak pressure gradient (62.1 ± 24.7 vs. 74.1 ± 34.6 mmHg; *p* = 0.04) and lower incidence of moderate-to-severe aortic regurgitation [AR] (30.4 vs. 55%; *p* = 0.04). However, no significant differences between both groups were noted in terms of AV mean pressure gradient and left ventricular measurements. In multivariate analysis, lower AV peak pressure gradient (OR = 0.97, 95% CI: 0.95–0.99) and absence of moderate-to-severe AR (OR = 0.65, 95% CI: 0.44–0.95) at baseline emerged as independent predictors of ViV-TAVI procedural success.

**Conclusion:** Valve-in-Valve TAVI for degenerated surgical valves is a feasible approach with high success rates, especially in those with lower AV peak pressure gradient and absence of moderate-to-severe AR. Studies with larger sample size and longer follow-up are required to further characterize the predictors of ViV-TAVI success and other clinical outcomes.

## Introduction

Valve-in-Valve transcatheter aortic valve implantation (ViV-TAVI) is increasing in popularity for patients with degenerated surgical valves (1). The Food and Drug Administration approved the procedure for high- and inoperable-risk patients, using the Medtronic and Sapien-3 valves in 2015 and 2017, respectively (2, 3). Several studies have shown high success rates for ViV-TAVI and a comparable safety profile to redo-surgery in terms of early and mid-term mortality, thromboembolic events, and permanent pacemaker implantation (4–6). A recent report from the PARTNER 2 ViV registry showed improved hemodynamics and excellent functional and quality of life outcomes for ViV-TAVI in high-risk patients (7). Therefore, the 2020 ACC/AHA guidelines have recommended ViV-TAVI for severely symptomatic patients with bioprosthetic valve stenosis or heart failure due to bioprosthetic valve regurgitation who are at high or prohibitive surgical risk (Class IIa recommendation) (8).

However, this procedure does not come without challenges. For a start, ViV-TAVI carries a higher risk of coronary obstruction (immediate and delayed) and patient-prosthesis mismatch than native TAVI. Second, the interaction between the original surgical and new transcatheter valves may increase the post-procedural pressure gradients (9). These challenges influence ViV-TAVI success rates and are often associated with worse clinical outcomes. However, they can be tackled by employing newer operative techniques, such as BASILICA approach, bioprosthetic valve fracture and improving patient selection for ViV-TAVI (10). Although many studies have evaluated the predictors of success and clinical outcomes of native valve TAVI (11–13), there is paucity of data on the determinants of ViV-TAVI procedural success in patients with degenerated surgical valves, to our knowledge.

Because the procedural success of ViV-TAVI is influenced by several variables, we performed the current analysis to investigate the predictors of in-hospital ViV-TAVI procedural success in patients with degenerated surgical valves. Our findings can improve patient selection and subsequently ViV-TAVI success rates in this patient population.

## Methods

### Patients

All consecutive patients who underwent ViV-TAVI for a degenerated surgical valve at the Cleveland Clinic Foundation (CCF) between January 2013 and December 2019 were analyzed. Data were extracted from our patient registries following a comprehensive chart review. Patients who underwent ViV-TAVI for a degenerated transcatheter valve (*n* = 55) or those with insufficient echocardiographic data to determine procedural success (*n* = 2) were excluded from the current analysis. The study protocol was approved by the Institutional Review Board at CCF.

### Data Collection

We obtained the following data from CCF patient charts: (A) Baseline patient data: Demographic, anthropometric, and laboratory values, as well as STS risk score and the presence of different cardiac and non-cardiac comorbidities, (B), Pre-operative echocardiographic data: left ventricular ejection fraction (LVEF%), end-systolic volume (LVESV: mL), end-diastolic volume (LVEDV: mL), end-systolic diameter (LVESD: cm), end-diastolic diameter (LVEDD: cm), left atrial volume (LA Vol. mL), posterior wall thickness (cm), aortic valve area (AVA: cm2), AV mean and peak gradients (mmHg), dimensionless Index (DI), and valvular regurgitation. (C) procedural characteristics: type of admission, anesthesia, TAVI access, surgical valve size, TAVI valve size and type, intra-operative complications, and employment of other techniques as pre/post-dilatation, sentinel device, Amplatz closure of paravalvular leakage. Patients with missing data in each variable were excluded from its respective analysis.

### Outcome Measure

Device success was defined according to the Standardized Endpoint Definitions for Transcatheter Aortic Valve Implantation Clinical Trials (14). In short, Device Success was defined as: (1) Successful vascular access, delivery, and deployment of the device and successful retrieval of the delivery system, (**2)** Correct position of the device in the proper anatomical location, (**3)** Intended performance of the prosthetic heart valve (aortic valve area >1.2 cm^2^ and mean aortic valve gradient <20 mmHg or peak velocity <3 m/s, without moderate or severe prosthetic valve regurgitation). Previous studies have defined procedural success as device success in the absence of major cardiovascular and cerebrovascular events (MACCE: Death, myocardial infarction, and stroke) during the peri-procedural period (11, 15). Because no periprocedural MACCE was recorded in our device success group, we considered these patients to have had procedural success.

### Statistical Analysis

Categorical data were expressed as frequency (%) and numerical data were expressed as median (interquartile range: IQR) or mean ± standard deviation where appropriate. The procedural success and failure groups were compared using Chi-Square and Fischer's Exact test (for categorical data) and independent sample *t*-test and Mann-Whitney test for numerical data. To identify the predictors of ViV-TAVI procedural success, we employed a multivariate logistic regression model. A *p* < 0.05 was considered statistically significant. All statistical analysis was conducted using SPSS (version 27 for Windows, IBM Inc., Armonk, NY).

## Results

### Baseline Characteristics

One hundred and eighty-six patients were analyzed in the current study: 165 (88.7%) in the procedural success and 21 (11.3%) in the failure groups. The method of determination of procedural failure in every patient is illustrated in Supplementary Table 1. Patients in the success group were significantly younger than those in the failure group (median: 70 vs. 78 years; *p* = 0.003). However, the distribution of gender, race, and anthropometric measures was comparable (*p* > 0.05). Further, the frequency of comorbidities, including diabetes mellitus, hypertension, coronary artery disease, atrial fibrillation, prior interventions (PCI, CABG, PPM, ICD) was similar between both groups (*p* > 0.05). The baseline characteristics of patients in the procedural success and failure groups are illustrated in Table 1.

**Table 1:** Baseline characteristics of patients in the procedural success and failure groups.

**Baseline characteristics**	**Procedural success** **(*****N*** **= 165)**	**Procedural failure** **(*****N*** **= 21)**	***P*** **-value**
Age (Years)	70 (61, 77)	78 (72, 83)	**0.003**
**Gender**			
Male	105 (63.6)	15 (71.4)	0.63
Female	60 (36.3)	6 (28.6)	
**Race**			
White	157 (95.2)	18 (85.7)	0.26
Non-White	8 (4.8)	3 (14.3)	
Height (cm) Mean ± SD	169.1 ± 10.6	171.6 ± 11.6	0.30
Weight (Kg)	77.1 (68.2, 89.8)	79.3 (69.2, 103)	0.52
STS risk score	6.3 (3.8, 8.3)	5.5 (3.8, 8.8)	0.71
Systolic blood pressure (mmHg)	134 (117.3, 145.8)	125 (113.5, 150.5)	0.51
Diastolic blood pressure (mmHg)	64 (57, 72.5)	61 (55.5, 67)	0.26
Hemoglobin (g/dL) Mean ± SD	11.7 ± 2.2	11.4 ± 2.2	0.57
Platelets (10^3^ cells/mL) Mean ± SD	179.8 ± 66.8	162.6 ± 37.3	0.25
Albumin (g/dL)	4 (3.6, 4.2)	4 (3.5, 4.4)	0.83
Bilirubin (mg/dL)	0.6 (0.5, 0.9)	0.7 (0.4, 1.1)	0.68
Creatinine (mg/dL)	1.2 (0.96, 1.5)	1.2 (0.8, 1.6)	0.72
BNP (pg/mL)	1,683 (816, 5,538)	2461 (674, 6,263)	0.65
**Comorbidities**			
Diabetes mellitus	44 (26.7)	9 (42.9)	0.12
Hypertension	148 (89.6)	18 (85.7)	0.48
Dyslipidemia	105 (63.6)	17 (80.9)	0.15
Coronary artery disease	83 (63.8)	12 (57.1)	0.63
Peripheral artery disease	80 (48.5)	9 (42.9)	0.65
Prior myocardial infarction	32 (19.3)	7 (33.3)	0.16
Prior stroke	20 (12.1)	3 (14.3)	0.73
Prior PCI	48 (29.1)	6 (28.6)	0.96
Prior CABG	70 (42.4)	11 (52.4)	0.48
Heart failure (Past 2 weeks)	83 (50.3)	15 (71.4)	0.07
Atrial fibrillation	90 (54.4)	11 (52.4)	0.85
Prior PPM implantation	33 (20)	5 (23.8)	0.77
Prior ICD implantation	14 (8.5)	4 (19)	0.12
Chronic kidney disease	55 (33.3)	10 (47.6)	0.23
Cancer	18 (10.9)	3 (14.3)	0.71

*Data are frequency (%) and median (Interquartile range) unless otherwise indicated. BNP, Brain naturitic peptide; ICD, Implantable cardioverter defibrillator; PPM, Permanent pacemaker implantation. P < 0.05 (Bold) are statistically significant*.

### Echocardiographic Parameters

The LVEF%, LVESV, LVEDV, AVA, and AV mean pressure gradient were comparable between both groups (*p* > 0.05). However, peak aortic valve gradient was significantly higher in the procedural failure than the success group (62.1 ± 24.7 and 74.1 ± 34.6 mmHg in the success and failure groups, respectively; *p* = 0.04). Moreover, the incidence of moderate-to-severe AR was significantly higher in the procedural failure group (30.4 vs. 55% in the success and failure groups, respectively; p = 0.04). The echocardiographic parameters of patients in the procedural success and failure groups are illustrated in Table 2.

**Table 2:** Echocardiographic parameters of patients in the procedural success and failure groups.

**Echocardiographic parameters**	**Procedural success** **(*N* = 165)**	**Procedural failure** **(*N* = 21)**	***P*** **-value**
LVEF (%)	55.7 (45.7, 62)	57.8 (35.8, 62.9)	0.99
LVESV (mL)	56.2 (33.5, 83.2)	57 (40.5, 148.8)	0.40
LVEDV (mL)	131.1 (84.6, 175.4)	143.8 (94.6, 227)	0.17
LVESD (cm)	3.6 (2.9, 4.4)	3.4 (3, 4.5)	0.98
LVEDD (cm)	5.1 (4.4, 5.7)	5.3 (4.6, 5.8)	0.31
LA Volume (mL)	88.4 (73, 111.7)	103.4 (81.2, 125.3)	0.11
Posterior wall thickness (cm)	1.2 (1, 1.3)	1.2 (1, 1.3)	0.86
**AV morphology**			
Bicuspid	52 (31.9)	6 (28.6)	0.75
Tricuspid	111 (68.1)	15 (71.4)	
AVA (cm^2^)	0.8 (0.7, 0.9)	0.5 (0.5, 0.6)	0.12
AV mean gradient (mmHg)	35.7 ± 15.8	42.6 ± 22.5	0.07
AV peak gradient (mmHg)	62.1 ± 24.7	74.1 ± 34.6	**0.04**
Dimensionless index	0.28 (0.22, 0.33)	0.22 (0.19, 0.29)	0.60
**Aortic regurgitation**			
None	11 (7)	1 (5)	**0.04**
Trivial	75 (47.4)	3 (15)	
Mild	24 (15.2)	5 (25)	
Moderate	24 (15.2)	7 (35)	
Severe	24 (15.2)	4 (20)	
**Mitral regurgitation**			
None	2 (1.3)	0 (0)	0.05
Trivial	87 (54.4)	9 (42.8)	
Mild	58 (36.2)	6 (28.6)	
Moderate	12 (7.5)	5 (23.8)	
Severe	1 (0.6)	1 (4.8)	
**Tricuspid regurgitation**			
None	5 (3)	1 (4.8)	0.05
Trivial	99 (60)	9 (42.8)	
Mild	44 (26.7)	5 (23.8)	
Moderate	11 (6.7)	5 (23.8)	
Severe	6 (3.6)	1 (4.8)	

*Data are frequency (%) and median (Interquartile range) unless otherwise indicated. LVEF, Left ventricular ejection fraction; LVESV, Left ventricular end-systolic volume; LVEDV, left ventricular end-diastolic volume; LVESD, Left-ventricular end-systolic diameter; LVEDD, left-ventricular end-diastolic diameter; AVA, Aortic valve area. P < 0.05 (Bold) are statistically significant*.

### Procedural Characteristics

The type of used anesthesia, the internal diameter of the degenerated surgical valve, TAVI valve size, type, and access site were comparable between both groups. Further, the incidence of intra-operative complications and length of hospital stay was comparable between both groups. However, a significant difference was noted in TAVI access site with higher utilization of the transfemoral route in the success group (97 vs. 90%, *p* = 0.01). Moreover, the success group had a higher utilization of the Sentinel cerebral protection system (47.3 vs. 23.8%, *p* = 0.04). The procedural characteristics of patients in the procedural success and failure groups are illustrated in Table 3.

**Table 3:** Procedural characteristics of patients in the procedural success and failure groups.

**Procedural characteristics**	**Procedural success** **(*N* = 165)**	**Procedural failure** **(*N* = 21)**	***P*** **-value**
**TAVI admission**			
Urgent	21 (12.7)	3 (14.3)	0.74
Elective	144 (87.3)	18 (85.7)	
Associated coronary intervention	7 (4.2)	1 (4.8)	0.91
Int. diameter (mm) of surgical valve	21 (19, 23)	20 (19, 23)	0.82
**Anesthesia used**			
Moderate sedation	138 (84.7)	14 (66.7)	0.1
General anesthesia	25 (15.3)	7 (33.3)	
**TAVI access type**			
Transfemoral	160 (97)	19 (90.4)	**0.01**
Transapical	5 (3)	1 (4.8)	
Transaxillary	0 (0)	1 (4.8)	
Sheath size used for TAVI delivery (mm)	14 (14,16)	14 (14,17)	0.33
TAVI valve size (mm)	23 (23, 26)	23 (23, 26)	0.70
**TAVI valve type**			
Balloon-expandable	137 (83)	16 (76.2)	0.44
Self-expandable	28 (17)	5 (23.8)	
Fluoroscopy time (min)	22.8 (15.8, 29.1)	28.6 (17.6, 35.9)	0.09
Contrast volume (ml)	20.5 (10, 37.3)	25 (15, 42)	0.52
Pre-dilatation	9 (5.4)	1 (4.8)	0.89
Post-dilatation	82 (49.7)	9 (42.8)	0.55
Sentinel device	78 (47.3)	5 (23.8)	**0.04**
PVL closure by Amplatz	3 (1.8)	1 (4.8)	0.38
Intra-operative complications	19 (11.5)	5 (23.8)	0.11
Length of hospital stay (day)	2 (1,6)	3 (2,12)	0.08

*Data are frequency (%) and median (Interquartile range). PVL, Paravalvular leakage; TAVI, transcatheter aortic valve implantation. P < 0.05 (Bold) are statistically significant*.

### Predictors of Procedural Success

In univariate regression analysis, younger age (OR = 0.88, 95% CI: 0.82–0.95, *p* = 0.01), lower AV peak pressure gradient before ViV-TAVI (OR = 0.96, 95% CI: 0.94–0.99, *p* = 0.04), larger diameter of the surgical valve (OR = 1.74, 95% CI: 1.32–3.62, *p* = 0.01), and absence of AR at baseline (OR = 0.66, 95% CI: 0.45–0.95, *p* = 0.02) predicted higher ViV-TAVI success rates.

Other echocardiographic parameters as LVEF (OR = 1, 95% CI: 0.97–1.04, *p* = 0.73), and AV mean pressure gradient (OR = 0.97, 95% CI: 0.95–1, *p* = 0.07), and operative characteristics as TAVI access site (OR = 0.31, 95% CI: 0.09–1.08, *p* = 0.06), TAVI valve size (OR = 0.99, 0.95% CI: 0.81–1.22, *p* = 0.98) and type (OR = 0.89, 95% CI: 0.69–1.15, *p* = 0.37), as well as using Sentinel device (OR = 1.14, 95% CI: 0.89-0.1.32, *p* = 0.08) did not reach statistical significance.

In multivariate regression analysis, only absence of moderate-to-severe AR at baseline (OR = 0.65, 95% CI: 0.44–0.95, *p* = 0.03) and lower AV peak pressure gradient (OR = 0.97, 95% CI: 0.95–0.99, *p* = 0.04) emerged as independent predictors of ViV-TAVI procedural success in patients with degenerated surgical valves.

## Discussion

The current analysis showed that ViV-TAVI for degenerated surgical valves has a high procedural success rate. In univariate analysis, younger age, lower AV peak pressure gradient, and absence of moderate-to-severe pre-operative AR could predict ViV-TAVI procedural success. However, only AV peak pressure gradient and absence of AR at baseline emerged as independent predictors in multivariate analysis. Our analysis is one of the largest published to date on ViV-TAVI (as an emerging procedure) for degenerated surgical vales.

The ViV-TAVI procedural success rate in our analysis was 88.7%. The reported success rates of ViV-TAVI in the literature vary between 52 and 100%. A recent meta-analysis by Mahmoud et al. pooled 24 studies (*N* = 5,294 patients) and reported an overall success rate of 97% (95% confidence interval: 94–98%), which is relatively higher than the current analysis. A significant heterogeneity was, however, observed that the authors attributed to differences in procedural volume and sample size between different studies. Further, the outcome definition of procedural success varies between studies. Interestingly, the same meta-analysis showed no significant difference in procedural success after subgrouping by valve type, which goes in line with our findings (16).

Several randomized trials and multicenter cohort studies have investigated the determinants of native valve TAVI procedural success, showing that existing comorbidities, echocardiographic findings (especially AV pressure gradients), type of the used valve, and employment of pre- and post-dilatation techniques are independent predictors of success (11–13). However, the same cannot be said for ViV-TAVI. The majority of published reports over the latter procedure are single-center observational studies. A recent meta-analysis of mostly small observational studies (<50 patients each) analyzed the predictors of 30-day and 1-year mortality in patients undergoing ViV-TAVI. The authors concluded that the independent predictors of 30-day mortality included study year (indicating improving operator experience), logistic EuroSCORE, and valve size <21 mm, while stenosis as a reason for the surgical valve failure was the only independent predictor of mortality at 1 year (17).

The predictors of procedural success in the present analysis were lower AV peak gradient and absence of moderate-to-severe AR before ViV-TAVI. The higher peak AV gradient may indicate further deterioration of the old surgical valve or worse morphological characteristics of the AV area, which may in turn affect the success of implanting the ViV-TAVI valve. Further, the higher peak gradient may predispose to significant regurgitation, which was another predictor in univariate regression. Considering that the margin of significance was narrow, this finding should be confirmed in randomized trials and larger observational studies. On the other hand, the mean AV pressure gradient did not show a significant predictive ability for procedural success in our analysis. The explanation for this finding could be statistical based on the width of data distribution. However, the relationship between the mean and peak AV pressure gradients needs further study.

In univariate analysis, younger age was an independent predictor for ViV-TAVI procedural success; however, it did not reach significance level in multivariate analysis. We are not aware of a ViV-TAVI study that evaluated the impact of age on procedural success. The aforementioned meta-analysis did not analyze the predictors for procedural success but did not identify age as an independent predictor for either 30-day or 1-year mortality after ViV-TAVI (17). For native valve TAVI, two studies by Buellesfeld et al. and Yamamoto et al. reported similar procedural success and short-term mortality outcomes in younger and older age groups (18, 19). The influence of age on ViV-TAVI procedural success, survival, and other clinical outcomes should be further studied in larger studies with longer follow-up durations.

The ViV-TAVI procedure is growing in popularity in patients with different risk groups. At our institution (CCF), we performed 11 ViV-TAVI procedures in 2013. This number consistently increased to 45 procedures in 2019. In the same time frame, 1599 redo-AVR surgeries were performed in our cardiothoracic surgery department. The decision for a patient with degenerated surgical valve to undergo ViV-TAVI or redo-AVR in our institution is made by a multidisiplinary team, taking into account several factors, including the patient's surgical risk and anatomical features. In the present study, we aimed to provide data to further guide this selection process and we believe that further investigation and integration of our findings in interventional practice can improve the outcomes of ViV-TAVI.

### Limitations

Although our analysis is one of the largest on ViV-TAVI to date and we used a standardized outcome definition for procedural success, our analysis has some limitations. First, the relatively small sample size (especially in the procedural failure group) may have masked the predictive potential of some parameters. Further, we only focused on the immediate outcome of ViV-TAVI. An analysis of long-term follow-up data on patient survival, valve performance, and other clinical outcomes is being planned from our institution. Randomized studies with large sample size and extended follow-up are warranted to investigate the predictors of ViV-TAVI success and compare clinical, hemodynamic, and quality of life on the long-term.

In conclusion, ViV-TAVI for degenerated surgical valves is a feasible approach with high success rates, especially in those with lower AV peak pressure gradient and absence of moderate-to-severe AR at baseline. Proper patient selection may improve the procedural outcomes of ViV-TAVI; however, more studies are needed to characterize optimal patient selection models and risk scores.

## Data Availability Statement

Data are available from the corresponding author on reasonable request.

## Ethics Statement

The studies involving human participants were reviewed and approved by Institutional Review Board at Cleveland Clinic Foundation. Written informed consent for participation was not required for this study in accordance with the national legislation and the institutional requirements.

## Author Contributions

AA and SK: idea conception. AA, OA, AS, and TI: data collection. AA and AS: data analysis. MF, JY, ZP, SS, RP, GR, AK, and SK: drafting the manuscript. All authors revised the final version and approved it for publication.

## Conflict of Interest

The authors declare that the research was conducted in the absence of any commercial or financial relationships that could be construed as a potential conflict of interest.

## Publisher's Note

All claims expressed in this article are solely those of the authors and do not necessarily represent those of their affiliated organizations, or those of the publisher, the editors and the reviewers. Any product that may be evaluated in this article, or claim that may be made by its manufacturer, is not guaranteed or endorsed by the publisher.

## Abbreviations

AR: Aortic regurgitation
ViV-TAVI: Valve-in-Valve transcatheter aortic valve implantation.
