# Supplementary material for: Predictors of Procedural Success in Patients With Degenerated Surgical Valves Undergoing Transcatheter Aortic Valve-in-Valve Implantation
Source: Front Cardiovasc Med. 2021 Sep 24;8:718835. doi: 10.3389/fcvm.2021.718835 (PMC8497979; doi:10.3389/fcvm.2021.718835)
Supplement: Supplementary file 1 [file Data_Sheet_1.PDF]

Supplementary Table 1: Determination of procedural Failure

Used Definition for device success:

- Successful vascular access, delivery, deployment, and retrieval.
- Correct position of the device in the proper anatomical location.
- Intended performance defined as
  - AVA > 1.2 and Mean gradient <20 mmHg OR
  - Peak Velocity <3m/s and no moderate-to-severe aortic regurgitation.

No.	Device Implantation Success	AVA <1.2 Cm^2	AV Mean Gradient <20 mmHg	Peak Velocity < 3m/sec	Moderate/severe regurgitation	Final Judgment
1	Yes	--	19	3.11	No	F
2	Yes	--	26	3.63	No	F
3	Yes	--	21	3.34	No	F
4	Yes	--	26	3.60	No	F
5	Yes	--	16	3.07	No	F
6	Yes	--	33	3.75	No	F
7	Yes	--	22	3.02	No	F
8	Yes	--	28	3.65	No	F
9	Yes	--	24	3.36	No	F
10	Yes	--	20	3.08	No	F
11	Yes		20	3.02	No	F
12	Yes	--	28	3.44	No	F
13	Yes	--	25	3.32	No	F
14	Yes		30	3.56	No	F
15	Yes		24	4.18	No	F
16	Yes	--	21	3.08	No	F
17	Yes	--	21	2.82	No	F
18	Yes	--	32	3.62	No	F
19	Yes	--	30	3.72	No	F
20	Yes	--	37	4.09	No	F
21	Yes	--	22	3.07	No	F
